# Draft Genome Sequence of Shewanella indica Isolated from a Marine Sponge (Callyspongia diffusa)

**DOI:** 10.1128/mra.00552-22

**Published:** 2022-09-29

**Authors:** Devika Raj, Abdulaziz Anas, Jasmin Chekidhenkuzhiyil

**Affiliations:** a CSIR-National Institute of Oceanography, Regional Centre Kochi, Kochi, India; Montana State University

## Abstract

The draft genome sequence of Shewanella indica strain MMRF542, which was isolated from a marine sponge (Callyspongia diffusa), is reported. The genome sequence provides insight into the ecological relevance and biotechnological potential of *Shewanella* species.

## ANNOUNCEMENT

*Shewanella* species are a physiologically diverse group of Gram-negative proteobacteria that are found in the aquatic ecosystem as free-swimming organisms or in association with sediment or other organisms. The abilities of *Shewanella* sp. strains to metabolize a diverse group of carbon sources and utilize different metals as anaerobic electron acceptors emphasize their ecological and biotechnological significance (1). The isolation of a *Shewanella* sp. strain from a marine sponge, its melanin production, and the potential of *Shewanella* melanin in recovering metals from liquid and protecting host cells from oxidative stress were reported in our previous studies (2, 3). By studying the whole genome, we aim to evaluate and infer the ecological relevance and biotechnological potential of *Shewanella* species. The *Shewanella* sp. strain was previously isolated from a marine sponge (Callyspongia diffusa) found in the coral reef ecosystem of the Gulf of Mannar, India, and was deposited in the Marine Microbial Reference Facility (MMRF) maintained at the CSIR-National Institute of Oceanography, Regional Centre Kochi (Kochi, India) (reference number MMRF542), and the National Centre for Microbial Resource (Pune, India) (accession number MCC0251). For the current work, the bacteria were retrieved from the lyophilized stock of MMRF542 in ZoBell marine broth, and the purity of the isolate was confirmed by Gram staining and sequencing of the 16S rRNA gene. *Shewanella* cells purified from a single colony were inoculated into ZoBell marine broth and incubated overnight at 24°C in a shaking incubator maintained at 100 rpm. The cells were separated by centrifugation at 8,000 rpm for 10 min, and DNA was extracted using the standard phenol-chloroform method without any modifications (4). The quality and purity of the DNA were assessed using agarose gel electrophoresis (on a 0.8% agarose gel) and spectrophotometry (absorbance at 260/280 nm in a NanoDrop ND-1000 spectrophotometer; Thermo Fisher Scientific), respectively, before samples were sent to the sequencing facility of Enfys Life Sciences Pvt. Ltd. DNA was sequenced to the requested depths using the Illumina HiSeq 4000 platform (2 × 150-bp reads) to generate paired-end reads. Briefly, paired-end libraries were prepared from an initial concentration of 100 ng of intact DNA using Illumina Nextera chemistry kits according to the manufacturer’s protocol, with IDT for Illumina 10-bp indices.

**FIG 1:**
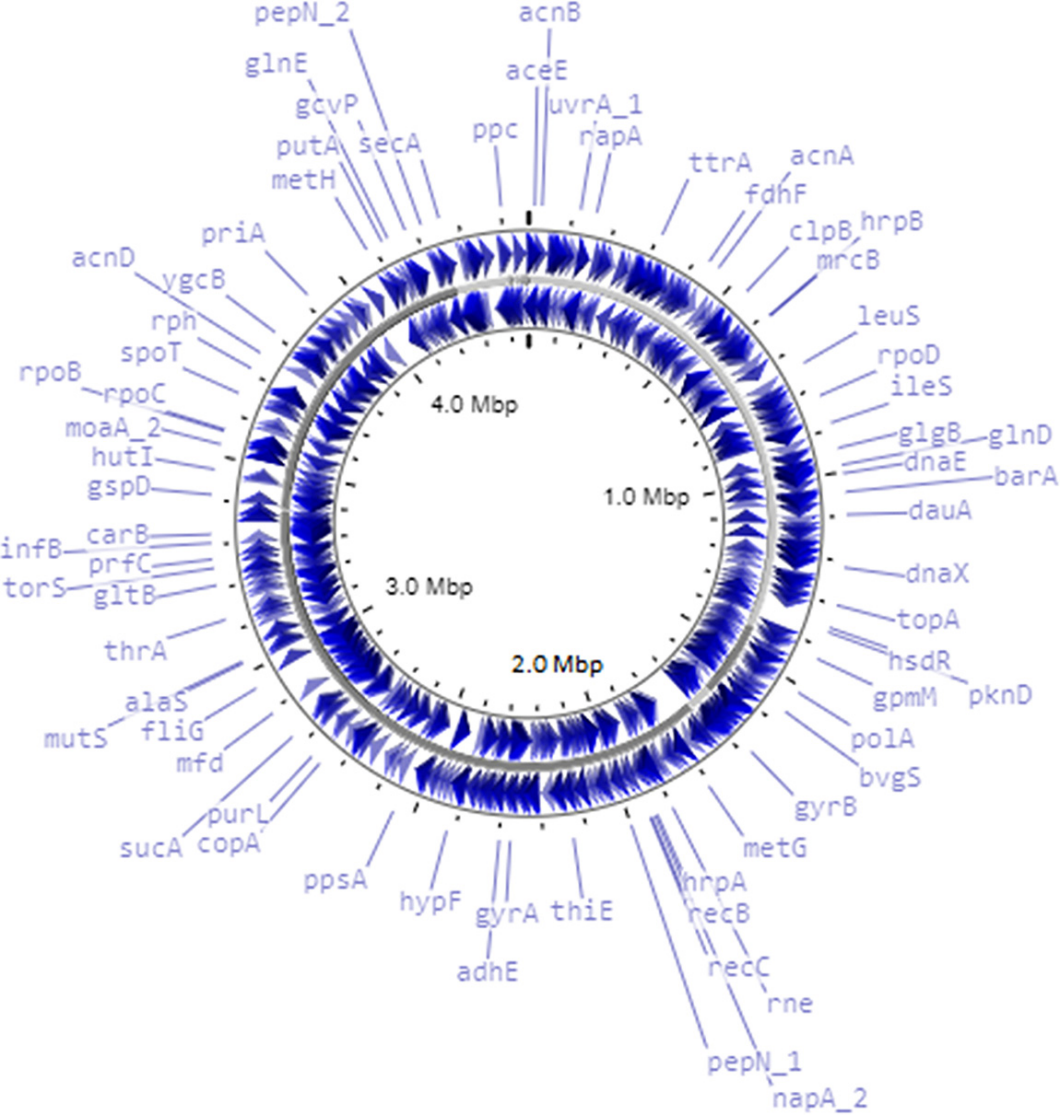
Genome map of Shewanella indica strain MMRF542, constructed using the CGView Server.

The resulting sequence consisted of 11 million reads, with a read length of 2 × 150 bp and coverage of 379×. Reads were examined with FastQC v0.11.9 (5), adapters were removed using TrimGalore v0.6.6 (6), and reads were trimmed with Trimmomatic v0.39 (7) and assembled with Unicycler v0.4.8 (8). The assembled contigs were taken for reference-guided secondary assembly with reference sequence Shewanella indica strain CNZ-1 (GenBank accession number NZ_QGDA01000001.1) using MeDuSa (9). The taxonomy of the organism was confirmed as Shewanella indica using PubMLST public databases for molecular typing and microbial genome diversity, with the *rpsA* gene (length of 1,668 bp) having 100% similarity (10). Genome annotation was performed with Prokka (11). Default software parameters were used throughout unless specified otherwise. The features of the whole-genome sequence of Shewanella indica strain MMRF542 are summarized in Table 1. The genome map was constructed using the CGView Server (12) (Fig. 1). The genome has a size of 4.4 Mbp, with a GC content of 52.2%. A total of 3,971 protein-coding sequences were detected by Prokka. The genome was found to harbor 4,062 genes in total, including 87 tRNA genes, 3 rRNA genes, 1 transfer-messenger RNA (tmRNA) gene, and 1 CRISPR sequence. In addition, we found genes of interest, namely, *tyrA, phhA, hppD*, and tyrosine aminotransferase genes, which are involved in pyomelanin production from homogentisic acid, in the organism. Shewanella indica strain MMRF542 requires in-depth research on its genomic features to gain complete insight into its mode of interaction with marine sponges and its role in protecting host cells from oxidative stress.

**TABLE 1:** Prokka genome annotation of Shewanella indica strain MMRF542

Feature	Finding for strain MMRF542
Genome size (Mbp)	4.4
GC content (%)	52.2
No. of contigs	235
No. of scaffolds	62
*N*_50_ (bp)	1,423,186
No. of coding sequences	3,971
No. of genes	4,062
No. of rRNA genes	3
No. of tRNA genes	87
No. of tmRNA genes	1
No. of CRISPR sequences	1

### Data availability.

The whole-genome sequence reads were deposited in DDBJ/ENA/GenBank under BioProject accession number PRJNA811810, BioSample accession number SAMN26363809, and SRA accession number SRR18190581. This whole-genome shotgun project was deposited in DDBJ/ENA/GenBank under accession number JALJEU000000000. The version described in this paper is version JALJEU010000000.
